# Mr. Charles Perks

**Published:** 1910-04-16

**Authors:** 


Mr. Charles Perks, of Burton-on-Trent, has died at the age of sixty-eight. In 1863 Mr. Perks became an L.S.A., and the following year gained the joint diploma. He had been a student at Queen's Hospital, Birmingham.

